# MARS: Microarray analysis, retrieval, and storage system

**DOI:** 10.1186/1471-2105-6-101

**Published:** 2005-04-18

**Authors:** Michael Maurer, Robert Molidor, Alexander Sturn, Juergen Hartler, Hubert Hackl, Gernot Stocker, Andreas Prokesch, Marcel Scheideler, Zlatko Trajanoski

**Affiliations:** 1 Institute for Genomics and Bioinformatics and Christian Doppler Laboratory for Genomics and Bioinformatics, Graz University of Technology, Petersgasse 14, 8010 Graz, Austria

## Abstract

**Background:**

Microarray analysis has become a widely used technique for the study of gene-expression patterns on a genomic scale. As more and more laboratories are adopting microarray technology, there is a need for powerful and easy to use microarray databases facilitating array fabrication, labeling, hybridization, and data analysis. The wealth of data generated by this high throughput approach renders adequate database and analysis tools crucial for the pursuit of insights into the transcriptomic behavior of cells.

**Results:**

MARS (Microarray Analysis and Retrieval System) provides a comprehensive MIAME supportive suite for storing, retrieving, and analyzing multi color microarray data. The system comprises a laboratory information management system (LIMS), a quality control management, as well as a sophisticated user management system. MARS is fully integrated into an analytical pipeline of microarray image analysis, normalization, gene expression clustering, and mapping of gene expression data onto biological pathways. The incorporation of ontologies and the use of MAGE-ML enables an export of studies stored in MARS to public repositories and other databases accepting these documents.

**Conclusion:**

We have developed an integrated system tailored to serve the specific needs of microarray based research projects using a unique fusion of Web based and standalone applications connected to the latest J2EE application server technology. The presented system is freely available for academic and non-profit institutions. More information can be found at .

## Background

Microarray analysis has become a widely used technique for the study of gene-expression patterns on a genomic scale [1,2]. Oligonucleotide and cDNA arrays have been utilized to study mRNA [3] and protein levels [4], to decipher protein-DNA interactions [5], to analyze the DNA copy number [6], to detect methylated sequences [7], and to analyze gene phenotypes in living mammalian cells [8]. Microarrays represent a very complex, multi step technique involving array fabrication, labeling, hybridization, and data analysis. Currently, most laboratories are using either one labeled sample (Affymetrix microarrays) or two labeled samples (cDNA microarrays) for hybridizations, but several applications have been established were three color microarrays are used [9,10]. State-of-the-art microarrays can have from several hundred up to tens of thousands of elements annotated by dozens of parameters. Information on details of the bench work, typically kept in lab notebooks or scattered files, as well as information regarding spotting, reliable tracking of the spotted molecules, scanning, and image quantification settings, is important for the computational analysis and reproducibility of experiments. Every step generates a wealth of data spanning tens of megabytes and in each of them errors may occur or protocols might need optimization to improve results. Moreover, all these information must be archived according to accepted scientific standards, which allow scientists to share common information and to make valid comparisons among experiments. For this reason the Microarray Gene Expression Data Society (MGED) [11] is focusing on establishing standards for microarray data annotation and exchange, facilitating the creation of microarray databases and related software implementing these standards. MGED is heavily promoting the sharing of high quality, well annotated data within the life sciences community. Their initiatives – MIAME (Minimum Information About a Microarray Experiment) [12], MGED Ontology [13], and MAGE-ML (MicroArray Gene Expression Markup Language) [14] – maximize the value of microarray data by permitting greater opportunities for sharing information within scientific groups and thus for discovery. These will ultimately affect the description, analysis, and management of all high throughput biological data.

The 'list of genes' resulting from microarray analysis is not the end of a microarray experiment. The major challenge is to assign biological function and to generate new hypotheses. The simplest way to find genes of potential biological interest is to search the normalized data for the highly expressed ones. Additionally, identifying patterns of gene expression and grouping genes into expression classes can provide greater insight into their biological relevance. For this purpose several supervised or unsupervised clustering algorithms like support vector machines (SVM), hierarchical clustering, k-means, self organizing map (SOM), or principal component analysis (PCA) are in use. The annotation of genes or gene clusters can be achieved by mapping them to the Gene Ontology (GO) [15] in order to provide insights into relevant molecular functions, biological processes, and cellular components [16]. Another way to identify genes of biological interest is to map the normalized data or gene expression clusters [17] to known metabolic pathways as provided e.g. by KEGG [18] or BioCarta [19].

Several academic as well as commercial systems are available that address at least some of the needs such as laboratory information management systems (LIMS) [20], microarray databases [21-24] and repositories, normalization, clustering, pathway or GO mapping tools or expression analysis platforms [25]. However, freely available systems which integrate all the aspects mentioned above are rare and may lack important issues like usability, scalability, or standardized interfaces. Furthermore, for such integrated systems it is desireable to use a uniform and state-of-the-art software architecture in order to enhance setup, maintenance and further development.

We have therefore developed a Microarray Analysis and Retrieval System (MARS) using latest Java 2 Platform, Enterprise Edtition (J2EE) software technology. MARS provides modules mandatory for microarray databases:

• a laboratory information management system (LIMS) to keep track of information that accrues during the microarray production and biomaterial manipulation

• MAGE-ML export of data for depositing to public repositories e.g. ArrayExpress [26], GEO [27]

For these components already existing projects [21,23,26] have been evaluated. Their advantages as well as disadvantages have been taken into account for the design of MARS. Widely used concepts have been taken into consideration and accepted standard libraries like MAGE-STK [11]have been used whenever possible. Additionally, we extented this solid foundation and added novel features which can be highlighted as distinct advantages of the MARS system.

• a quality management application storing necessary quality control parameters indispensable for high-quality microarray data

• Web services to connect several well established tools such as normalization, clustering and pathway annotation applications

• applications for microarray normalization, gene expression clustering, and pathway exploration that are tightly integrated into the microarray analysis pipeline

• a novel, comprehensive, and Web based user management system to administrate institutes, groups, users, and their corresponding access rights

## Implementation

### Software architecture

**Figure 1:**
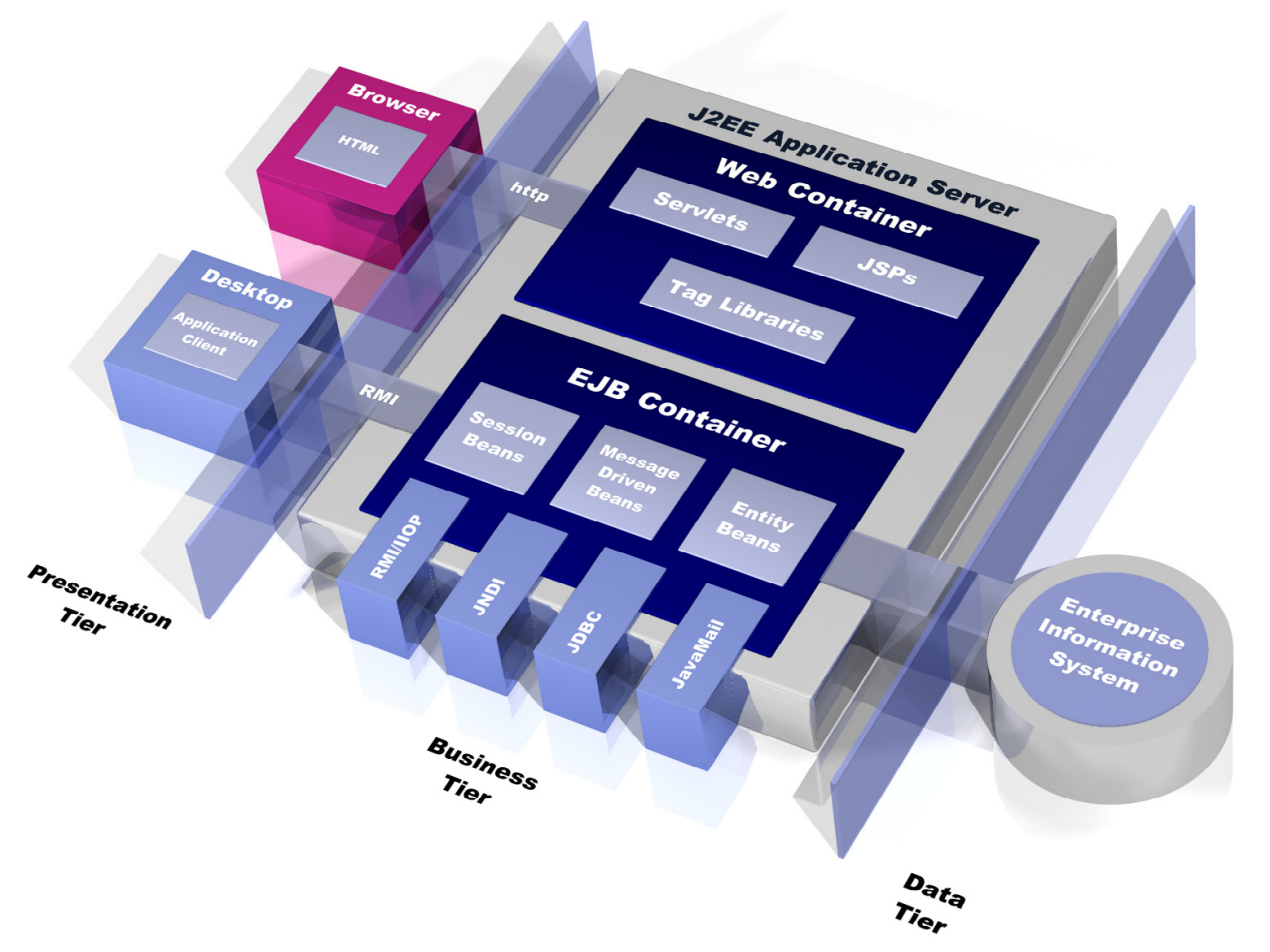
**Three tier Java 2 Enterprise Edition software architecture**. The J2EE platform simplifies the development of enterprise applications by providing standardized modular components like EJBs, JSP and Servlets. Furthermore it is providing a complete set of services to those components.

MARS is based on a three tier architecture (Figure 1) using the Java 2 Platform, Enterprise Edition (J2EE), which defines a standard for developing multi tier enterprise applications. The J2EE platform simplifies the development of enterprise applications using on standardized, modular components like Enterprise JavaBeans (EJB), Java Servlets, Java Server Pages (JSP), and XML technology.

A relational database (Oracle or PostgreSQL) builds the data- or Enterprise Information System tier. In the middle tier the J2EE compliant application server JBoss [28] is situated. It manages the access to the relational database as well as the interaction with the data. The Web server in conjunction with a servlet-container is responsible for the presentation tier. All the servlets and JSPs are executed to enable input and output of an application and to manage the applications workflow logic. An advantage of a multi tier architecture is that different tiers can be deployed to different servers, enabling load distribution as well as scalability.

### Systems

The database schema, the business logic, and the Web interface can be subdivided into five major groups:

#### 1. Microarray production

To address the needs of many laboratories which produce their own microarrays, MARS includes a generic array production LIMS. It manages data regarding the substances (clones) and their localization in microtiter plates, the array design spotted on the support, as well as single arrays and array batches. The flexible and generic database design facilitates mapping of the steadily changing laboratory workflow. Additionally, each plate can be assigned to a library, which designates the organism and contains details about the cloning vector, forward and reverse primer and standard molecule annotations including gene name, accession number, UniGene number, and sequence. Substances stored in microtiter plates may undergo certain manipulations such as PCR amplification. Therefore a PCR amplification event can be assigned to a plasmid plate in order to generate a PCR plate in the database.

After entering the information necessary for spotting, a file is generated and prepared for download. This file is used by the spotting robot software to generate an array design file. After the spotting run has been completed, the array design file has to be uploaded into MARS. For each spotting run an array batch has to be created in MARS, and all slides spotted by this spotting run have to be assigned to this array batch. Additionally, important parameters regarding the spotting run such as temperature, duration, or humidity can be assigned to this array batch. Barcode tracking is employed for plates as well as for arrays to reduce possible input errors. Laboratories using commercial arrays have to upload the array design instead and define an array batch afterwards.

#### 2. Sample preparation

Samples can be annotated in a user-customizable manner. MARS allows the annotation of biological descriptions such as the source and characteristics of a sample (e.g. tissue and disease), any genetic and chemical manipulation and stimulation. Performing such annotations in free text fields leads to large undefined vocabularies and makes them difficult to query. Thus, three different annotation types are provided: 1) enumeration enabling the usage of defined vocabularies or ontologies, 2) numbers to allow scoring and counting and 3) free text. Annotated samples will be linked to an extract, enabling a lab worker to annotate the extraction method, protocol, concentration, purity, and quantity. The labeled extract stores information on used extract quantity, the label and the labeling protocol.

#### 3. Hybridization and raw data management

The hybridization page archives parameters regarding the hybridization tool and method and is linked to the used labeled extracts. In contrast to several other microarray databases MARS can handle any number of labeled extracts and thus allows the storage of multi color experiments. Resulting images from hybridized scanned slides can be uploaded to MARS and added to a hybridization record. It is noteworthy that a hybridization can have several image sets associated with images of different scanner settings. After analyzing the images several different raw datasets analyzed with different program settings can be uploaded and added to the appropriate image set.

#### 4. Experiment annotation

**Figure 2:**
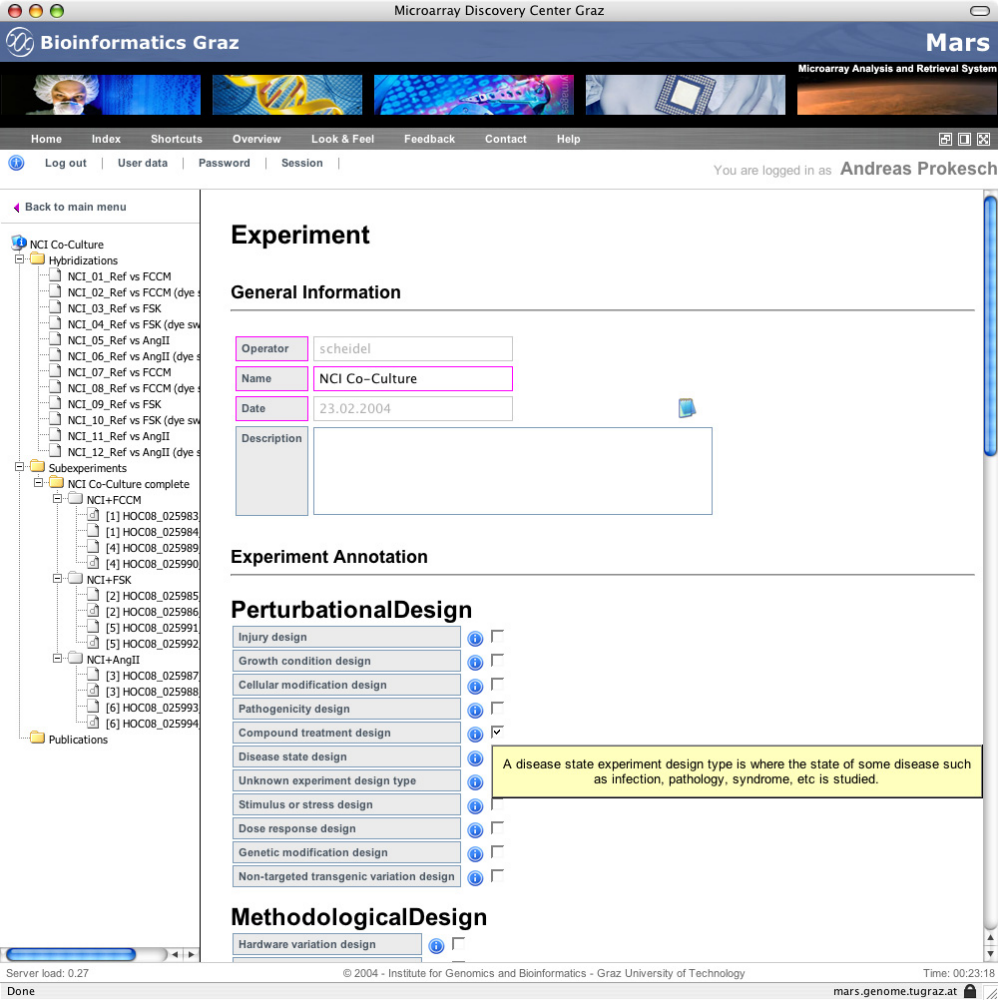
**Experiment annotation**. Web interface to define microarray experiments according to the MGED Ontology.

A set of hybridizations forms an experiment. To store the experimental design these hybridizations can be divided into classes, paired, and flagged as a dyeswap hybridization. Additionally, an experiment can be annotated using MGED Ontology definitions (Figure 2) to specify the perturbational, methodological, and epidemiological design, as well as the biological properties. Transformed datasets can be added to classes and their corresponding raw dataset.

#### 5. Quality management

**Figure 3:**
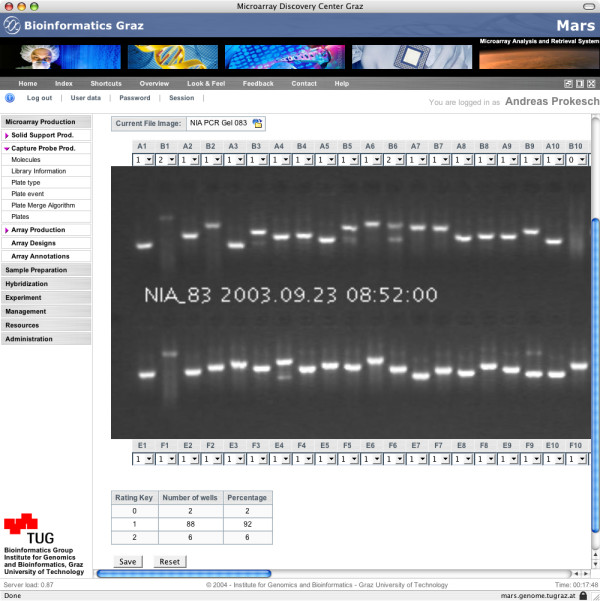
**Quality control**. A gel image from PCR products can be scored and associated to a plate.

To ensure high quality data and to allow the detection of possible sources of errors, a powerful quality management system has been integrated into MARS. This system is based on standard quality control procedures conducted during microarray production as well as during sample preparation, extraction and hybridization. In order to control the quality of PCR and purified PCR products generated during probe production, authorized users can upload gel images and analyze the bands according to a predefined schema (Figure 3). Based on this schema, PCR products can be identified later as a source of bad or missing spots on a slide. Quality annotation can be viewed by any user.

**Figure 4:**
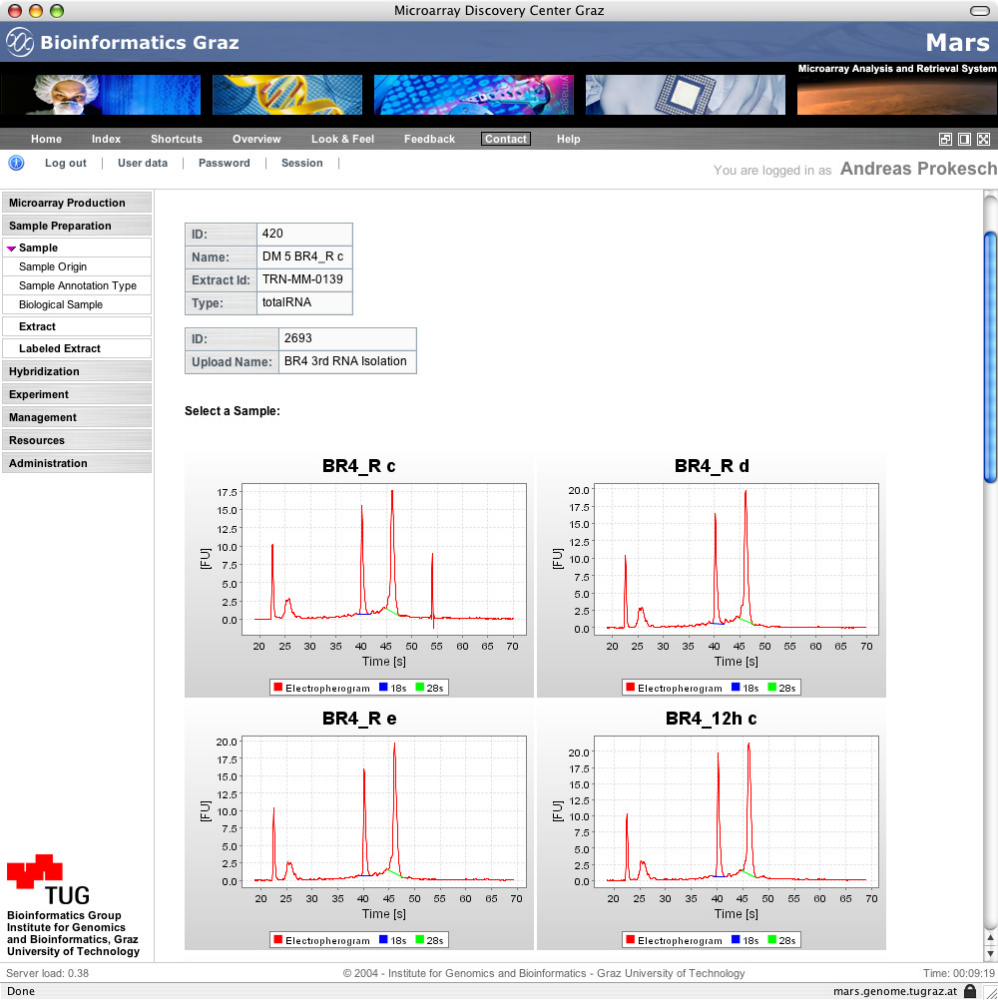
**Quality control**. Bioanalyzer analysis to check the RNA quality for a given RNA extract.

Slides can be scanned after fixation and/or after staining and parameters like spot walking or the number of missing spots are used to determine slide quality. In addition to array production quality controls, it is also necessary to check the quality of samples and its extracts. Data gained from an Agilent Bioanalyzer or gel images can be uploaded and analyzed either automatically (Bioanalyzer file) or manually (gel images) (Figure 4). Labeled extracts can be measured with a spectrophotometer to assess the efficiency of dye incorporation. Results of these measurements can be entered into MARS and the corresponding efficiency is calculated automatically. Finally, the quality of a hybridized slide is analyzed by extracting and displaying several statistical parameters from the raw data result file and by examining positive and negative controls printed onto a slide.

### Data interfaces

One of the most important parts for the acceptance of a database is the data import interface. To allow the import of generic file formats, we have implemented a user definable parser that allows to read any tab delimited text file. The user has to define a file format where file columns are assigned to appropriate database fields. MARS allows to define file formats for importing plates, raw datasets, transformed datasets, and array designs.

Any file that has to be imported, linked, or used has to be uploaded to MARS at first. Afterwards these data can be analyzed by the users at their office desk without having to use another central storage system. Uploaded files are stored on the servers file system where MARS has been installed. Additionally, links to these files are maintained in the relational database to prevent the deletion of already imported, linked, or used files.

The implementation of other Web based applications and more important, the usage and correct linkage of their stored data have been addressed by an External Application Connector Interface. Additional applications like supplementary quality checks can be added without any additional coding in MARS. The MARS user interface is dynamically displaying links to all former registered applications.

The Microarray Gene Expression Markup Language (MAGE-ML) has emerged as a language to describe and exchange information about microarray based experiments [29]. MAGE-ML is based on XML (eXtensible Markup Language) and can describe microarray designs, microarray manufacturing information, microarray experiment setup and execution information, gene expression data, and data analysis results. By using the Java MAGE-STK (Mage Software Toolkit) [11] MARS is able to export samples, extracts, labeled extracts, arraydesigns, raw datasets, or whole experiments including several hybridizations.

### Web service

In order to grant users access to MARS with software they are familiar with (e.g. BioConductor [30] or Matlab [31]), MARS provides a well defined Simple Object Access Protocol (SOAP) interface. SOAP is an XML-based communication protocol and encoding format for inter-application communication. After minor software adaptions these interfaces allow to authenticate against MARS, to browse own and shared datasets, to download raw data, to filter the data, and to insert transformed datasets into MARS. To take advantage of the SOAP Web service we provide a Java library called MARSExplorer, that allows software developers to extend their programs with data access functionality to MARS. Additionally, if no firewall is located between the client software and MARS, the MARS API (Application Programming Interface) can be used to access public accessible methods via the RMI (Remote Method Invocation) interface.

### Access control

To avoid unauthorized database access in a multi user environment the control of user access is a crucial criterion for the acceptance of any database managing functional genomic data. Furthermore, the definition of several fine grained user access levels that allow to visualize, edit or delete data (e.g. expression and sample data, protocols) based on the user rights is mandatory. Therefore we have developed an extensible and easy to use authentication and authorization system (AAS) which rests upon the same technology as MARS. In addition to its Web based management interface, the AAS provides software libraries that enable existing and new applications the integration of highly sophisticated authentication and authorization mechanisms. Moreover, the AAS provides single-sign-on to all its connected Web based applications. Since this AAS can also be used in various projects or institutions relying upon freely available software, MySQL has been choosen as database management system. If desired, this AAS can also manage Windows and Unix accounts using SAMBA [32] and LDAP (Lightweight Directory Access Protocol) [33]. For instance, at the Insitute for Genomics and Bioinformatics all Web based applications and user accounts are administrated by one single instance of the AAS.

## Results

### Database

**Figure 5:**
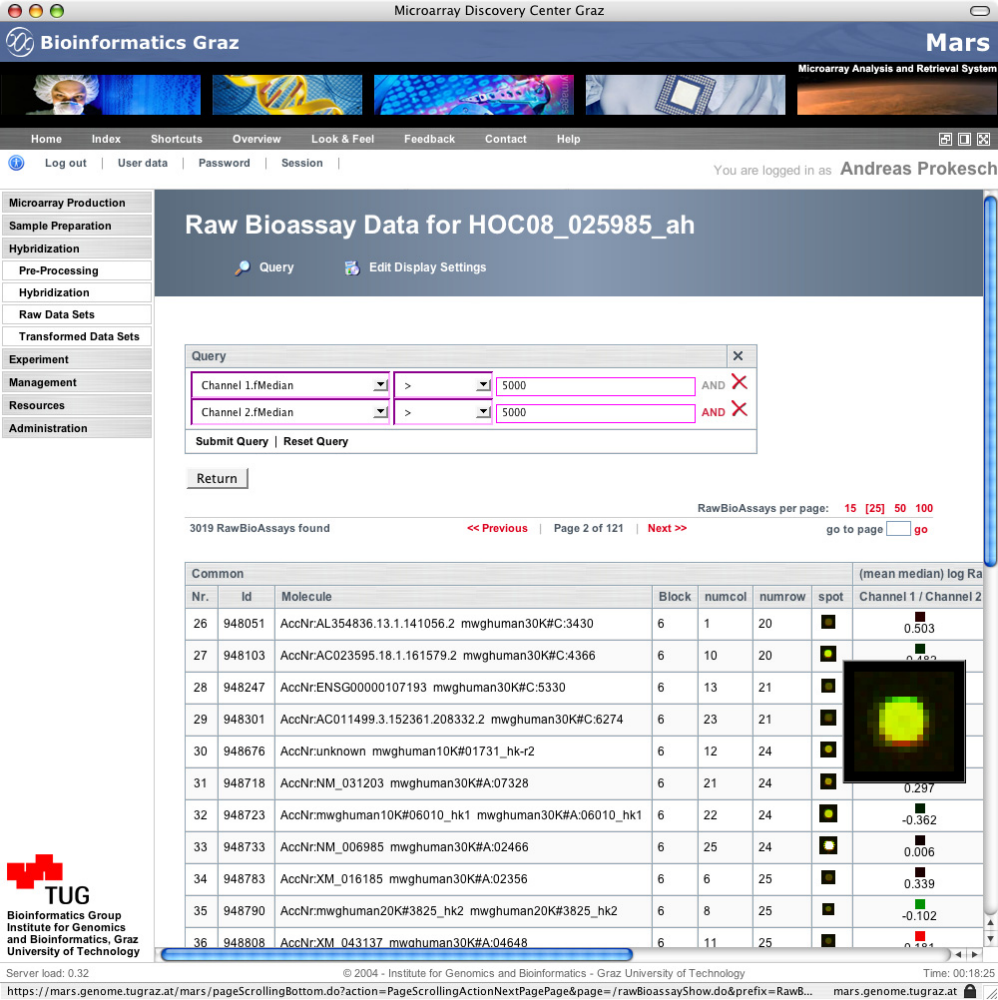
**Typical MARS interface listing stored records**. It allows to query for specific records using the user friendly query tool.

All MARS user interfaces are providing a consistent look and feel and are very intuitive to use. In general, the Web based user interface can be divided into two types of user interaction pages: The first one is an input form, where a user can record required and optional data according to the MIAME standard. Required fields are marked in magenta and are validated for correct input. The second allows to list all stored records. To keep the information on a page simple, a user can hide unnecessary datafields. Furthermore it is possible to query for specific records (Figure 5) using the MARS report query tool. Because all Web pages are linked together, MARS permits to follow all conducted steps from the transformed data back to the corresponding well in a microtiter plate and to visualize the results of quality controls. The description of an experiment including hybridizations and their raw datasets is typically the starting point for further analysis.

### Analytical pipeline

**Figure 6:**
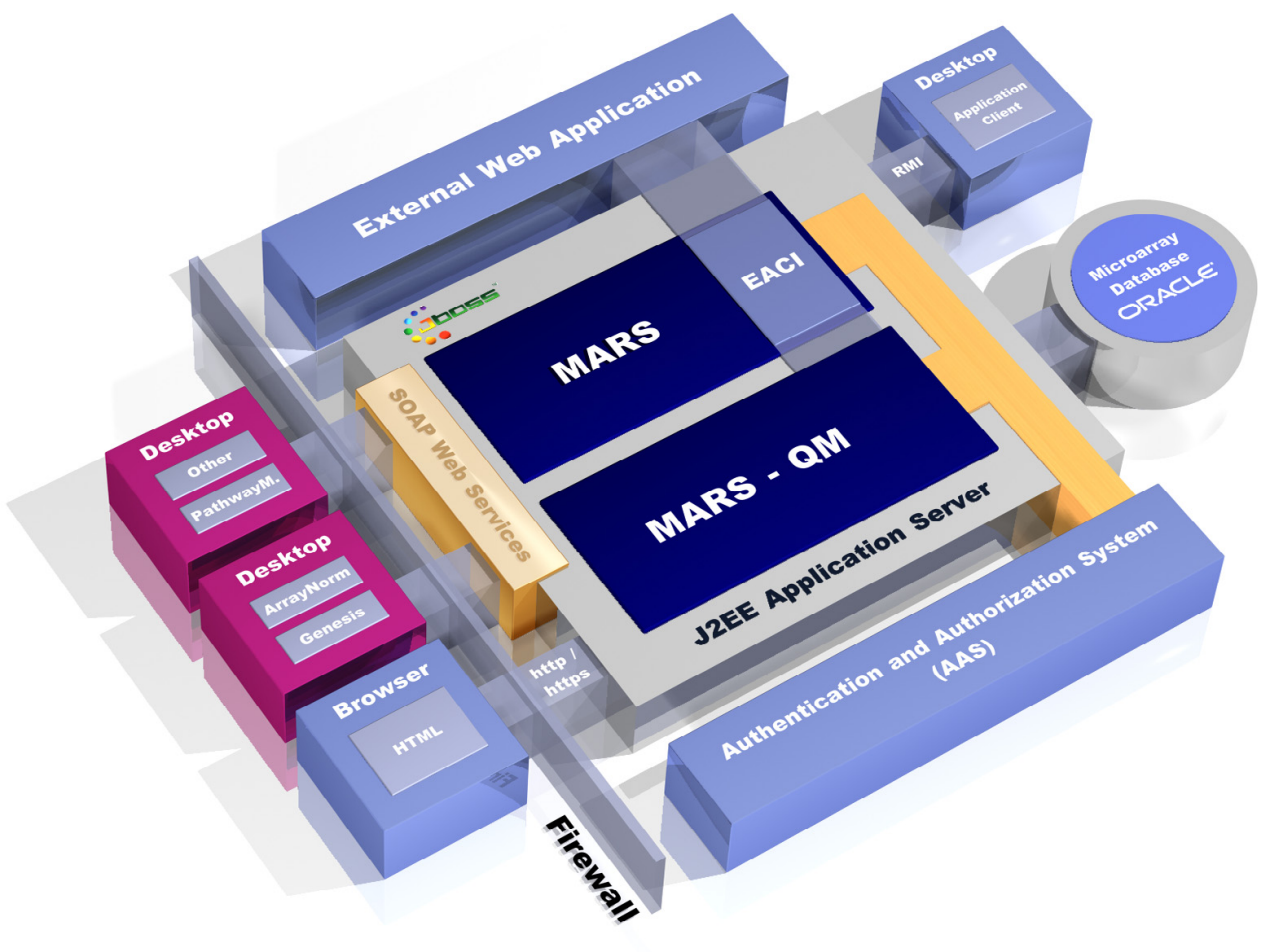
**MARS system interactions**. MARS and MARS-QM are deployed in a J2EE compliant application server. Interaction is possible either with a standard Web browser or an application supporting the SOAP or RMI protocol. The External Application Connector Interface (EACI) facilitates to connect to data from additional Web applications. SOAP and http/https enable MARS access also through firewalls.

The usability of MARS and the functionality of the provided interfaces and APIs (Figure 6) are revealed by the integration of MARS into an analytical pipeline of microarray analysis, beginning with image analysis, normalization, gene expression clustering, and finally mapping of gene expression data onto biological pathways.

After entering all required information into MARS, the first step is to normalize the raw data gathered from the image analysis software in order to remove systematic and random errors inherent in the data. ArrayNorm [34], an application for visualization, normalization and analysis of two-color microarray data facilitates these essential steps. Raw data including the definition of experiment classes (biological conditions) and pairs (replicated or dye swapped slides) from whole experiments can be loaded from MARS into ArrayNorm. After visualization and applying different normalization methods like linear regression, LOWESS, or self-normalization, the transformed intensities can be written back to MARS, including the history of the applied methods. The next step in the analytical pipeline is usually gene expression cluster analysis to extract the fundamental patterns inherent in the data and to organize genes with similar expression patterns into biological relevant clusters. Normalized gene expression data can be loaded into Genesis [35]. Genesis allows to cluster the dataset using various similarity distance measurements and different clustering algorithms like hierarchical clustering, k-means, self-organizing maps, principal component analysis, correspondence analysis, and support vector machines. Moreover it is possible to perform one-way ANOVA to identify differentially expressed genes and to incorporate the Gene Ontology (GO) to map gene expression clusters to GO terms. Results can be written back into MARS.

Finally, the Pathway Editor [36] provides the opportunity to access MARS and to map data either from whole experiments or from gene expression clusters to specified pathways in order to get an overview of gene expression changes and their influencing factors. All aforementioned applications have integrated MARSExplorer to connect to MARS and to query, up- and download datasets.

## Discussion

The database design, state-of-the-art software technology, well designed user interface, and its application interfaces make MARS a powerful tool for storing, retrieving, and analyzing multi color microarray data. The fusion of Web based and standalone applications provides researchers with an unique set of computational tools for genomic and transriptomic data.

The main strengths of MARS are:

### 1. Data interfaces

Fundamental for the acceptance of a database are the data interfaces. In principle two types of data interfaces for human computer interactions can be distinguished. Standalone applications allow better program-user interactions while having the drawback that several or even very old versions are in use. On the other hand Web based applications can be easily used on every computer without any installation effort and they provide the same and newest version to all users with the cost of limited user interaction. To ensure data integration and good usability we have developed the core data manipulation and storing functions using Web based technology and for data analysis we are using robust applications.

### 2. Application interfaces

Excellent usability does not only account for primely data interfaces. The ability to easily import data and the availability of well defined application interfaces are also crucial. Different institutions use diverse, mostly self tailored applications with proprietary and varying data formats. MARS provides several data and application interfaces. To import data we provide user definable and manageable parsers. When a user is uploading data, MARS tries to find an appropriate parser based on the file data or format header. Once the data is uploaded and stored, the data can be analyzed using the provided applications. For scientists who would like to analyze their data with other software, MARS provides also a Web service data interface. After some slight adaptations, users can authenticated and down- or upload data. Providing a Web service interface allows through its wide spread and platform independence to be implemented in all well-established programming languages and in tools like Matlab or BioConductor.

Existing Web applications can be plugged-in using the EACI that enables the linkage between data provided by the plugged-in application and data stored in MARS. Moreover it is possible to extend MARS without having to amend the MARS source code.

### 3. Quality management

In order to assure high-quality data and to understand or optimize lower value data it is important to be able to trace back all conducted quality control steps. MARS traces several quality measurements performed during the microarray production as well as during the sample preparation, extraction, and hybridization process. These quality checks are implemented as an additional application called MARS-QM, which is tightly integrated into MARS.

### 4. Data sharing and export

MARS enables users to share their datasets with other users. Supplementary to the user oriented data management an institution oriented level has been introduced. This amelioration allows several institutes to store their data into one data repository without having to share common settings and resources such as scanners, but offering the possibility to share the data among them.

Besides the sharing of microarray experiment data we provide the possibility to export hybridizations and experiments using the common exchange format MAGE-ML. This feature facilitates the easy sharing and publishing of high quality, well annotated data within the life sciences community by uploading the generated files to public repositories like ArrayExpress [26].

### 5. User management

Since microarray- as well as the corresponding quality control data may contain highly sensitive data, we have integrated our AAS into MARS to provide authentication and fine grained authorization mechanisms. The combination of AAS and External Application Connector Interface provides through a single-sign-on mechanisms and dynamic linkage of data the possibility to assemble heterogeneous Web applications to one powerful suite.

Because information attached to molecules is changing quickly, we are currently implementing the possibility to update and enhance the information tagged to a molecule. Changing this information on the molecule level may affect already existing results. In order to avoid such precarious alterations, a user should be able to update the molecule information for each experiment separately instead of replacing the initial molecule information. Further ongoing projects concentrate on the integration of Affymetrix GeneChip arrays into MARS and the improvement of MAGE-ML export capabilities in order to obtain approval from the ArrayExpress annotation team. Both features will be made available to the public in the next major release.

## Conclusion

In summary, we have developed an integrated system consisting of a microarray database and a microarray quality control database, that has been tailored to serve the specific needs of microarray based research projects. Due to the unique fusion of using Web based and standalone applications connected to the latest J2EE application server technology, bioinformatics researchers receive the benefits of standards-based software engineering. The system can provide a model how to build up a similar platform for other emerging functional genomics technologies.

## Availability and requirements

• Project name: MARS

• Project home page: 

• Operating system: Solaris, Linux, Windows

• Programming language: Java, HTML

• Other requirements: Java JDK 1.4.x, Oracle 9i, MySQL 4.0.xx, Server with at least 1 GBytes of main memory

• License: IGB-TUG Software License

• Any restrictions to use by non-academics: no

Installation of MARS is not complicated and should be manageable within a few hours if necessary access rights especially to Oracle and MySQL are granted. Step-by-step instructions are provided at the projects Web site together with the files and scripts necessary for installation. The reference installation of MARS is running on a Sun Fire V880 server under Solaris 9 using Oracle 9i as Database Management System. Attached is a Storage Area Network (SAN) with 2 TBytes.

The production instance of MARS contains information from more than 1000 microtiter plates, 24 array batches, 232 hybridizations, and 312 rawbioassays with about 9,170,000 datapoints.

## Authors' contributions

MM, RM, and AS designed and implemented the current version of MARS. They were responsible for the database design, the development of the business- as well as presentation logic. JH developed the quality management system and incorporated it into MARS. MM and JH were the lead developers of the AAS. HH, AP, GS, and MS have been involved in the compilation of the user requirements document and contributed to the conception and design of the system. ZT was responsible for the overall conception and project coordination. All authors gave final approval of the version to be published.

## References

[B1] Yang IV, Chen E, Hasseman JP, Liang W, Frank BC, Wang S, Sharov V, Saeed AI, White J, Li J, Lee NH, Yeatman TJ, Quackenbush J (2002). Within the fold: assessing differential expression measures and reproducibility in microarray assays. Genome Biol.

[B2] Schena M, Shalon D, Heller R, Chai A, Brown PO, Davis RW (1996). Parallel human genome analysis: microarray-based expression monitoring of 1000 genes. Proc Natl Acad Sci U S A.

[B3] Schena M, Shalon D, Davis RW, Brown PO (1995). Quantitative monitoring of gene expression patterns with a complementary DNA microarray. Science.

[B4] Haab BB, Dunham MJ, Brown PO (2001). Protein microarrays for highly parallel detection and quantitation of specific proteins and antibodies in complex solutions. Genome Biol.

[B5] Iyer VR, Horak CE, Scafe CS, Botstein D, Snyder M, Brown PO (2001). Genomic binding sites of the yeast cell-cycle transcription factors SBF and MBF. Nature.

[B6] Pollack JR, Perou CM, Alizadeh AA, Eisen MB, Pergamenschikov A, Williams CF, Jeffrey SS, Botstein D, Brown PO (1999). Genome-wide analysis of DNA copy-number changes using cDNA microarrays. Nat Genet.

[B7] Yan H, Park SH, Finkelstein G, Reif JH, LaBean TH (2003). DNA-templated self-assembly of protein arrays and highly conductive nanowires. Science.

[B8] Mousses S, Caplen NJ, Cornelison R, Weaver D, Basik M, Hautaniemi S, Elkahloun AG, Lotufo RA, Choudary A, Dougherty ER, Suh E, Kallioniemi O (2003). RNAi Microarray Analysis in Cultured Mammalian Cells. Genome Res.

[B9] Hessner MJ, Wang X, Khan S, Meyer L, Schlicht M, Tackes J, Datta MW, Jacob HJ, Ghosh S (2003). Use of a three-color cDNA microarray platform to measure and control support-bound probe for improved data quality and reproducibility. Nucleic Acids Res.

[B10] Tsangaris GT, Botsonis A, Politis I, Tzortzatou-Stathopoulou F (2002). Evaluation of cadmium-induced transcriptome alterations by three color cDNA labeling microarray analysis on a T-cell line. Toxicology.

[B11] MGED – Microarray Gene Expression Data Society Home Page. http://www.mged.org.

[B12] Brazma A, Hingamp P, Quackenbush J, Sherlock G, Spellman P, Stoeckert C, Aach J, Ansorge W, Ball CA, Causton HC, Gaasterland T, Glenisson P, Holstege FC, Kim IF, Markowitz V, Matese JC, Parkinson H, Robinson A, Sarkans U, Schulze-Kremer S, Stewart J, Taylor R, Vilo J, Vingron M (2001). Minimum information about a microarray experiment (MIAME)-toward standards for microarray data. Nat Genet.

[B13] Stoeckert CJ, Causton HC, Ball CA (2002). Microarray databases: standards and ontologies. Nat Genet.

[B14] Spellman PT, Miller M, Stewart J, Troup C, Sarkans U, Chervitz S, Bernhart D, Sherlock G, Ball C, Lepage M, Swiatek M, Marks WL, Goncalves J, Markel S, Iordan D, Shojatalab M, Pizarro A, White J, Hubley R, Deutsch E, Senger M, Aronow BJ, Robinson A, Bassett D, Stoeckert CJ, Brazma A (2002). Design and implementation of microarray gene expression markup language (MAGE-ML). Genome Biol.

[B15] Gene C Ontology (2001). Creating the gene ontology resource: design and implementation. Genome Res.

[B16] Pasquier C, Girardot F, Jevardat dFK, Christen R (2004). THEA: ontology-driven analysis of microarray data. Bioinformatics.

[B17] Mlecnik B, Scheideler M, Hackl H, Hartler J, Sanchez-Cabo F, Trajanoski Z (2005). PathwayExplorer: web service for visualizing highthroughput expression data on biological pathways. Nucleic Acids Res.

[B18] Kanehisa M, Goto S, Kawashima S, Nakaya A (2002). The KEGG databases at GenomeNet. Nucleic Acids Res.

[B19] BioCarta – Charting Pathways of Life. http://www.biocarta.com.

[B20] Kokocinski F, Wrobel G, Hahn M, Lichter P (2003). QuickLIMS: facilitating the data management for DNA-microarray fabrication. Bioinformatics.

[B21] Saal LH, Troein C, Vallon-Christersson J, Gruvberger S, Borg A, Peterson C (2002). BioArray Software Environment (BASE): a platform for comprehensive management and analysis of microarray data. Genome Biol.

[B22] Gardiner-Garden M, Littlejohn TG (2001). A comparison of microarray databases. Brief Bioinform.

[B23] Killion PJ, Sherlock G, Iyer VR (2003). The Longhorn Array Database (LAD): An Open-Source, MIAME compliant implementation of the Stanford Microarray Database (SMD). BMC Bioinformatics.

[B24] Gollub J, Ball CA, Binkley G, Demeter J, Finkelstein DB, Hebert JM, Hernandez-Boussard T, Jin H, Kaloper M, Matese JC, Schroeder M, Brown PO, Botstein D, Sherlock G (2003). The Stanford Microarray Database: data access and quality assessment tools. Nucleic Acids Res.

[B25] Theilhaber J, Ulyanov A, Malanthara A, Cole J, Xu D, Nahf R, Heuer M, Brockel C, Bushnell S (2004). GECKO: a complete large-scale gene expression analysis platform. BMC Bioinformatics.

[B26] Brazma A, Parkinson H, Sarkans U, Shojatalab M, Vilo J, Abeygunawardena N, Holloway E, Kapushesky M, Kemmeren P, Lara GG, Oezcimen A, Rocca-Serra P, Sansone SA (2003). ArrayExpress – a public repository for microarray gene expression data at the EBI. Nucleic Acids Res.

[B27] Edgar R, Domrachev M, Lash AE (2002). Gene Expression Omnibus: NCBI gene expression and hybridization array data repository. Nucleic Acids Res.

[B28] JBoss.com:: The Professional Open Source Company. http://www.jboss.org.

[B29] Quackenbush J (2004). Data standards for 'omic' science. Nat Biotechnol.

[B30] Dudoit S, Fridlyand J (2003). Bagging to improve the accuracy of a clustering procedure. Bioinformatics.

[B31] The MathWorks – Matlab and Simulink for Technical Computing. http://www.mathworks.com.

[B32] Samba – opening windows to a wider world. http://www.samba.org.

[B33] OpendLDAP. http://www.openldap.org.

[B34] Pieler R, Sanchez-Cabo F, Hackl H, Thallinger GG, Trajanoski Z (2004). ArrayNorm: comprehensive normalization and analysis of microarray data. Bioinformatics.

[B35] Sturn A, Quackenbush J, Trajanoski Z (2002). Genesis: cluster analysis of microarray data. Bioinformatics.

[B36] Trost E, Hackl H, Maurer M, Trajanoski Z (2003). Java editor for biological pathways. Bioinformatics.

